# Immune transgene-dependent myocarditis in macaques after systemic administration of adeno-associated virus expressing human acid alpha-glucosidase

**DOI:** 10.3389/fimmu.2023.1094279

**Published:** 2023-03-22

**Authors:** Juliette Hordeaux, Ali Ramezani, Steve Tuske, Nickita Mehta, Chunjuan Song, Anna Lynch, Katherine Lupino, Jessica A. Chichester, Elizabeth L. Buza, Cecilia Dyer, Hongwei Yu, Peter Bell, Jill M. Weimer, Hung Do, James M. Wilson

**Affiliations:** ^1^ Gene Therapy Program, Department of Medicine, Perelman School of Medicine, University of Pennsylvania, Philadelphia, PA, United States; ^2^ Amicus Therapeutics, Inc., Philadelphia, PA, United States

**Keywords:** AAV, toxicity, cytotoxic, T cell, Pompe disease

## Abstract

Immune responses to human non-self transgenes can present challenges in preclinical studies of adeno-associated virus (AAV) gene therapy candidates in nonhuman primates. Although anti-transgene immune responses are usually mild and non-adverse, they can confound pharmacological readouts and complicate translation of results between species. We developed a gene therapy candidate for Pompe disease consisting of AAVhu68, a clade F AAV closely related to AAV9, that expresses an engineered human acid-alpha glucosidase (hGAA) tagged with an insulin-like growth factor 2 variant (vIGF2) peptide for enhanced cell uptake. Rhesus macaques were administered an intravenous dose of 1x10^13^ genome copies (GC)/kg, 5x10^13^ GC/kg, or 1 x 10^14^ GC/kg of AAVhu68.vIGF2.hGAA. Some unusually severe adaptive immune responses to hGAA presented, albeit with a high degree of variability between animals. Anti-hGAA responses ranged from absent to severe cytotoxic T-cell-mediated myocarditis with elevated troponin I levels. Cardiac toxicity was not dose dependent and affected five out of eleven animals. Upon further investigation, we identified an association between toxicity and a major histocompatibility complex class I haplotype (Mamu-A002.01) in three of these animals. An immunodominant peptide located in the C-terminal region of hGAA was subsequently identified *via* enzyme-linked immunospot epitope mapping. Another notable observation in this preclinical safety study cohort pertained to the achievement of robust and safe gene transfer upon intravenous administration of 5x10^13^ GC/kg in one animal with a low pre-existing neutralizing anti-capsid antibodies titer (1:20). Collectively, these findings may have significant implications for gene therapy inclusion criteria.

## Introduction

Immune responses to adeno-associated virus (AAV) vector capsids can potentially reduce patient inclusion and limit gene therapy efficacy through pre-existing neutralizing antibodies (NAbs) and cytotoxic T-cell responses, respectively (1, 2). Immune responses to the transgene product can also limit the safety and efficacy of gene therapies when cytotoxic responses to non-self epitopes occur in patients with null mutations or large deletions of the gene being replaced (3, 4). This emergent safety concern was recently highlighted in several AAV-micro-dystrophin trials in which some patients with large dystrophin deletions developed immune-mediated myositis and myocarditis 24–42 days after dosing. The adverse events likely arose from T-cell-mediated responses directed against the dystrophin region for which the patients were cross-reactive immune-material-negative, leading to epitopes being recognized as non-self (5). In the absence of proven immune suppression protocols that achieve long-term tolerance to non-self-transgene epitopes, these trials typically exclude patients with null and/or large mutations, further narrowing inclusion criteria already limited by the titer of pre-existing antibodies to the AAV capsid (6).

When using nonhuman primates (NHPs) to evaluate the safety of an AAV gene therapy product for human patients, immune responses to the non-self-transgene product are frequently observed, although they are typically minimal and non-adverse due to the high degree of conserved coding sequences between NHPs and humans. Secreted proteins expressed from transgenes typically elicit a non-adverse humoral immune response with anti-transgene product antibodies that limit the ability to detect the transgene product in blood samples, thus confounding pharmacological readouts (7–10). Non-conserved reporter transgenes such as green fluorescent protein (GFP) lead to rapid clearance of transgene from transduced tissues in NHPs, whereas rodents do not mount cytotoxic responses to GFP (11). Some therapeutic transgenes can trigger similar T-cell cytotoxic responses in NHPs that have the potential to clear transduced cells and cause adverse inflammatory reactions in target organs, although this is less common (12, 13). It remains unknown why some transgenes lead to tolerization or moderate humoral responses while others are eliminated by robust cytotoxic responses. Moreover, predicting such outcomes is difficult as they depend on a complex interplay between the inherent immunogenicity of a transgene product and the host’s major histocompatibility complex (MHC) class I genotype. There are known examples of associations between drug-induced hypersensitivity reactions and macaque MHC haplotypes in the field of small-molecule drugs (14). Researchers should incorporate similar efforts to document MHC genotypes whenever adverse reactions following gene therapy occur in preclinical and clinical studies.

We aimed to develop a gene therapy for Pompe disease using systemic (intravenous/IV) or combined intra-cisterna magna (ICM) and systemic AAVhu68 encoding an engineered variant insulin-like growth factor 2 (vIGF2) peptide-tagged human acid alpha-glucosidase (hGAA). The vIGF2 peptide tag was strategically engineered for improved selective binding to the intended IGF2/cation-independent mannose-6-phosphate receptor (IGF2/CI-MPR) while greatly diminishing interactions with related receptors and IGF binding proteins. vIGF2 peptide was shown to maintain high-affinity binding to IGF2/CI-MPR with substantially reduced binding to the insulin and insulin-like growth factor 1 (IGF-1) receptors, thus enhancing its cross-correction potential while limiting the risk of hypoglycemia and other potential off-target effects (15, 16). The rationale for a dual IV and ICM arm was to target muscles (IV) and motor neurons (ICM) concurrently to rescue both peripheral and central components of Pompe disease related to neuromuscular pathology (17, 18). After showing promising efficacy in a mouse model of Pompe disease (19), we studied the pharmacology and safety of AAVhu68.vIGF2.hGAA in NHPs. When conducting pilot pharmacology and safety studies in rhesus macaques, we observed atypical adaptive responses to hGAA that varied widely between animals, ranging from no response to severe T-lymphocyte-rich myocarditis with elevated troponin I levels. Therefore, we aimed to further characterize the T-cell responses by studying interferon-gamma enzyme-linked immunospot (ELISPOT) responses toward the capsid and transgene peptides. To elucidate the possible causes of this high inter-individual variability, we genotyped the NHP cohort’s MHC alleles to identify possible associations with cardiac toxicity.

Another aim of these studies was to document the impact of pre-existing neutralizing AAV antibodies on gene transfer. Two of eleven macaques that received vector had pre-existing NAbs to the AAV capsid at titers of 1:20 and 1:160, respectively, while the remaining macaques had undetectable levels (<1:5). We investigated gene transfer efficacy and safety and compared our findings with results obtained in NAb-negative animals.

## Materials and methods

All data discussed in the article are available in the main text or **Supplementary Materials**. Due to the small number of animals within each treatment group (n=2), only descriptive data are presented and no statistical analyses were performed.

### Study design

**Table S1** presents our study design. Briefly, we aimed to investigate the pharmacology and pilot safety of our test article in NHPs. We administered the vector intravenously (IV) to healthy rhesus macaques (*Macaca mulatta*) at a dose of 1x10^13^ or 5x10^13^ genome copies (GC)/kg of AAVhu68.vIGF2.hGAA. Two groups of animals received both IV and ICM vector injections at 5x10^13^ GC/kg and 3x10^13^ GC, or 1x10^14^ GC/kg and 1x10^13^ GC, respectively (n=2 animals per route and per dose; **Table S1**). None of the eight animals had any detectable pre-existing NAbs. To determine the effects of pre-existing anti-AAV NAbs, three additional animals with increasing levels of pre-existing NAb (screening NAb titers of <1:5, 1:10, and 1:80; n=3; **Table S1**) received an IV dose of 5x10^13^ GC/kg. All animals in this study survived until their scheduled necropsy time point at 60 days post-vector administration.

### Test article

The test article consisted of a serotype hu68 AAV (AAVhu68) vector expressing a codon-optimized hGAA transgene N-terminally tagged with vIGF2 under the control of a CAG promoter/enhancer combination with a rabbit beta-globulin polyadenylation signal (referred to as AAVhu68.vIGF2.hGAA). To investigate test-article-related findings with neuronal degeneration in dorsal root ganglia (DRG), we utilized a DRG-detargeted construct [same expression cassette with four tandem repeats of miR183 target sites in the 3’ untranslated region, as described in (20)] in three animals (**Table S1**). The expression construct was flanked by AAV serotype 2 inverted terminal repeats and was cloned in a plasmid containing a kanamycin resistance gene for manufacturing. pAAV2/hu68 was provided by the Penn Vector Core (RRID: SCR_022432). The vector was produced by triple transfection of adherent HEK293 cells and purified by affinity chromatography *via* a POROS™ CaptureSelect™ AAVX resin, followed by anion exchange chromatography. We titrated the purified vectors with droplet digital polymerase chain reaction (ddPCR) using primers targeting the rabbit beta-globin polyA sequence, as previously described (21).

### Animals and test article administration

We obtained rhesus macaques (*Macaca Mulatta*) of Chinese origin from Covance, Orient Bioresources or Primgen. All animal procedures were approved by the Institutional Animal Care and Use Committee of the University of Pennsylvania or at the Children’s Hospital of Philadelphia (CHOP). We housed the animals in an Association for Assessment and Accreditation of Laboratory Animal Care International-accredited Nonhuman Primate Research Program facility at the University of Pennsylvania or CHOP in stainless steel squeeze-back cages. Animals received varied enrichments such as food treats, visual and auditory stimuli, manipulatives, and social interactions. The NAb negative animals were randomized to receive either an IV low dose of 1x10^13^ GC/kg, an IV high dose of 5x10^13^ GC/kg, an IV high dose of 5x10^13^ GC/kg plus an ICM high dose of 3x10^13^ GC, or an IV very high dose of 1x10^14^ GC/kg plus an ICM low dose of 1x10^13^ GC. The NAb-positive animals were assigned to the IV high dose group only. For IV administration, we diluted the vector in sterile 1x Dulbecco’s phosphate-buffered saline (PBS) and administered the vector *via* a catheter placed in the saphenous vein. The NHPs received vector diluted in a volume of 1 mL of artificial cerebrospinal fluid injected into the cisterna magna under fluoroscopic guidance, as previously described (22).

### Clinical pathology

Serum chemistry, hematology, and coagulation and troponin I measurements were performed by the contract facility Antech GLP or Antech Diagnostics (Morrisville, NC). Complement factors were analyzed by the National Jewish Health Complement Laboratory.

### Immunology

We measured peripheral blood T-cell responses against hGAA and the AAVhu68 capsid using IFN-γ ELISPOT assays according to previously published methods (11, 23) using peptide libraries specific for the AAVhu68 capsid and hGAA transgene. Positive response criteria were >55 spot-forming units per 10^6^ lymphocytes and three times the medium negative control upon no stimulation. In addition, we assayed T-cell responses in lymphocytes extracted from livers after necropsy on study day 60. We used liver lymphocytes for further epitope mapping in two animals with robust responses by dividing the hGAA peptide libraries into sub-pools containing fewer peptides and stimulated cells until the positive response was narrowed down to single peptides.

MHC genotyping was performed by the Wisconsin National Primate Research Center utilizing the Illumina MiSeq next-generation sequencing platform to obtain short amplicon genotyping focused on the most polymorphic segments of MHC genes, as previously described (24).

We measured NAbs against the AAVhu68 capsid in serum using an *in vitro* HEK293-cell-based assay, as previously described (6, 23). NAb titers are reported as the reciprocal of the sample dilution that inhibits transduction in 50% of the cells. The limit of detection of the assay was 1:5 sample dilution. We also measured antibody titers to hGAA in serum by ELISA. Briefly, plates were coated with recombinant hGAA overnight at 4°C, blocked, and incubated with serial dilutions of NHP plasma. Presence of anti hGAA antibodies was revealed using anti-Monkey goat IgG conjugated with horseradish peroxidase (Thermo Fisher Scientific, PA1-84631) diluted at 1:1000 in block buffer. Plates were read at absorbance 450nm and data analyzed in Excel to determine titer.

We performed dual immunofluorescence (CD3 + CD20, CD3 + CD8, CD4 + FoxP3) on formalin-fixed, paraffin-embedded heart sections using a signal amplification system from Akoya Biosciences (Marlborough, MA; Opal 4-Color Manual IHC Kit). Sections were deparaffinized through a xylene and ethanol series and then sequentially subjected to first antigen retrieval (13 min of microwave radiation in citrate buffer), incubation with the first primary antibody followed by a corresponding secondary peroxidase-labeled antibody, and staining with the first Opal dye. The sections were then subjected to a second antigen retrieval, incubation with the second primary antibody followed by a corresponding secondary peroxidase-labeled antibody, and staining with a second Opal dye. All antibodies were diluted in 1% donkey serum in PBS containing 0.2% Triton. After staining, we mounted the sections in ProLong Gold Antifade mounting medium (ThermoFisher) containing 4′,6-diamidino-2-phenylindole to stain the nuclei. We used the following primary antibodies: rabbit anti-CD3 (Dako A0452, 1:1,000 dilution), rabbit anti-CD20 (ThermoFisher Pa5-16701, 1:2,500 dilution), rabbit anti-CD8 (Abcam ab4055, 1:500 dilution), mouse anti-CD4 (LSBio LS-B9816, 1:200 dilution), and rabbit anti-FoxP3 (Abcam Ab99962, 1:200 dilution).

### Biodistribution

DNA was extracted from tissues using QIAamp Mini Extraction kits (Qiagen). We performed biodistribution analysis by TaqMan quantitative PCR targeting the vector polyadenylation signal sequence. Assay results are reported as GC/diploid genome (DG).

### Histology

Tissues were fixed in formalin, embedded in paraffin, sectioned, and stained with hematoxylin and eosin according to standard protocols. A board-certified veterinary anatomic pathologist (ELB) performed histological evaluations of the tissues. Lesions were scored on a severity scale from 0 (within normal limits) to 5 (severe lesion, affecting approximately 95% of the tissue on a section), and DRG pathology was scored as previously published (25).

### hGAA expression

We performed hGAA immunostaining on sections from formalin-fixed, paraffin-embedded tissue samples. Sections were deparaffinized with xylene and ethanol, heat-treated under pressure with citrate-based antigen unmasking solution (Vector Laboratories), and then treated sequentially with 2% H_2_O_2_ (15 min; Sigma), avidin/biotin blocking reagents (15 min each; Vector Laboratories), and blocking buffer (1 h; 5% donkey serum in PBS + 0.3% Triton), followed by incubation with primary and biotinylated secondary antibody. We used a rabbit antibody against hGAA as the primary antibody (Sigma HPA029126, diluted 1:100 in 1% donkey serum in PBS with 0.2% Triton; overnight incubation at 4°C) followed by biotinylated donkey anti-rabbit antibody (Jackson Immuno Research 711-065-152, diluted 1:750 in 1% donkey serum in PBS with 0.2% Triton; incubation for 30 min at room temperature). We employed a Vectastain Elite ABC kit (Vector Laboratories) with 3,3’-diaminobenzidine as a substrate to visualize bound antibodies as a brown precipitate. Sections were slightly counterstained with hematoxylin to show nuclei. We scanned whole slides using a bright field scanner (Aperio AT2) and quantified the positive signal *via* VIS version 2019.07.0.6328 (Visiopharm, Hoersholm, Denmark) with thresholding using the “HDAB-Hematoxylin” classification feature.

We measured GAA enzyme activity, resulting from both endogenous macaque GAA and hGAA transgene product, in plasma using a sensitive fluorogenic assay, as previously described (26).

## Results

### Pharmacology in NHPs following AAV-hGAA gene transfer

We previously assessed the *in vivo* efficacy of AAVhu68.vIGF2.hGAA in a *Gaa* knockout mouse model of Pompe disease (19). To investigate the pharmacology and pilot safety in NHPs, the vector was IV administered to two groups of healthy Rhesus macaques (*Macaca mulatta*) at a dose of 1x10^13^ or 5x10^13^ genome copies (GC)/kg. Another two groups of animals received both IV and ICM injections of 5x10^13^ GC/kg and 3x10^13^ GC, or 1x10^14^ GC/kg and 1x10^13^ GC, respectively (n=2 animals per route and per dose; **Table S1**). All eight animals had undetectable pre-existing NAb levels. To determine the effects of pre-existing anti-AAV NAbs, three additional animals with increasing pre-existing NAb titers (NAb titers pre-dosing were <1:5, 1:10 to 1:20, and 1:80 to 1:160; n=3; **Table S1**) each received 5x10^13^ GC/kg IV. All animals in this study survived until their scheduled necropsy time point at days 60 post-vector administration.

The vector genome biodistribution in tissues showed dose-dependent gene transfer in NAb-negative animals with transgene product detected in the diaphragm, skeletal muscles, and heart in all IV-dosed animals, as well as in spinal cord lower motor neurons in animals receiving dual IV and ICM administration (**Table S2**, **Figure S1**). We observed the highest transduction levels in the liver (ranging from 12.04 GC/DG at 1x10^13^ GC/kg IV to 144.62 GC/DG at 5x10^13^ GC/kg), followed by the heart (ranging from 0.14 GC/DG at 1x10^13^ GC/kg IV to 2.81 GC/DG at 5x10^13^ GC/kg) and the spleen (ranging from 0.07 GC/DG at 1x10^13^ GC/kg IV to 1.14 GC/DG at 5x10^13^ GC/kg). Skeletal muscle exhibited lower gene transfer levels, with values ranging from 0.01 GC/DG at the low dose to 0.63 GC/DG at the higher dose (**Table S2**). Biodistribution results in the animal with the low NAb titer of 1:20 were similar to those of the NAb-negative animals. However, transduction levels were significantly lower in the liver, heart, and skeletal muscles, but increased in the spleen of the animal with the high 1:160 NAb titer (**Table S2**). We confirmed efficient gene transfer in the low-NAb-titer animal after IV administration of 5x10^13^ GC/kg at the protein level in the heart *via* immunohistochemistry staining and whole-slide quantification (**Figure S2**). The observation of robust liver, cardiac, and skeletal muscle transduction in the animal that had a low NAb titer of 1:20 suggests that high-dose IV AAV can overcome low levels of NAbs to achieve gene transfer efficacy similar to that in NAb-negative animals.

**Figure 1:**
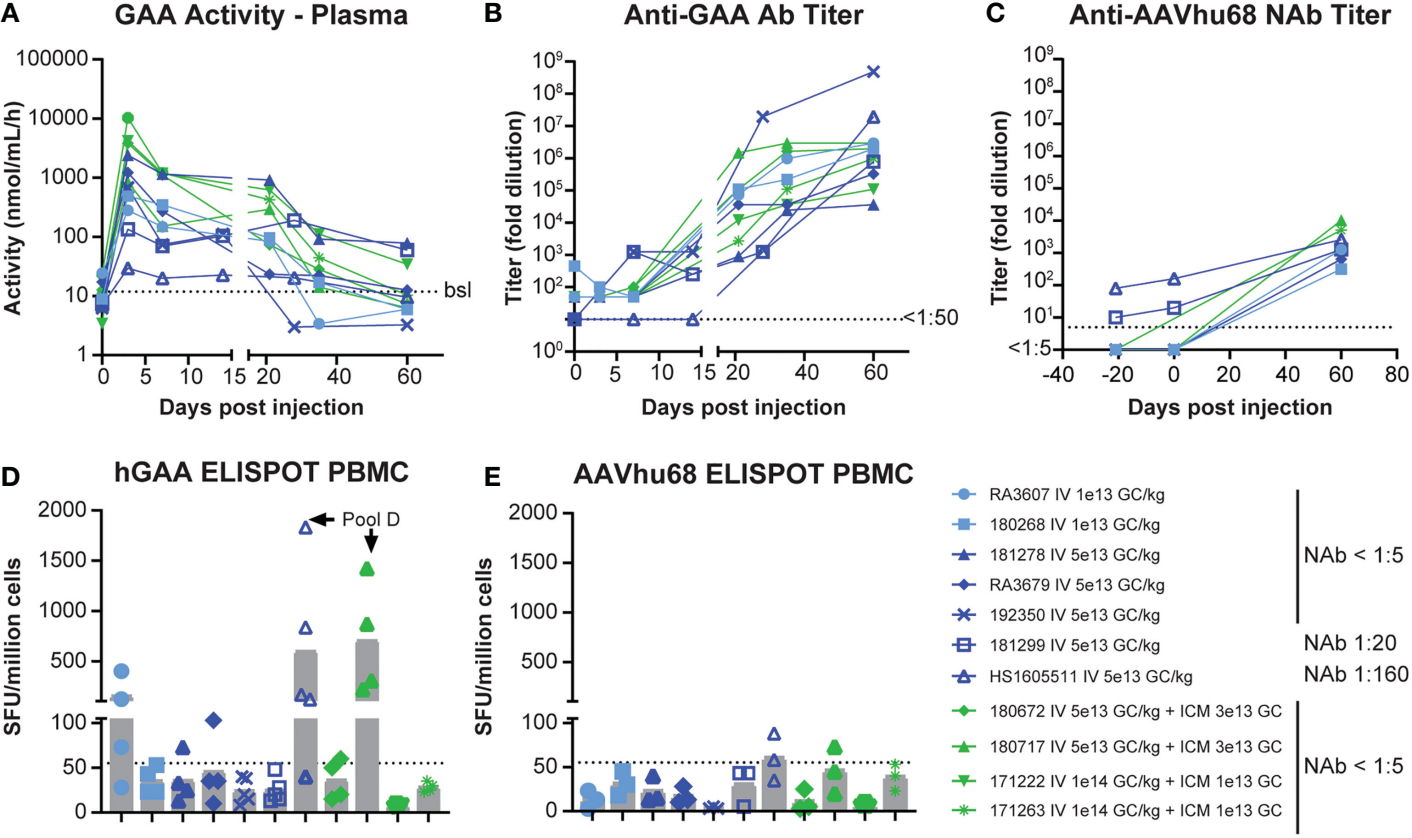
Pharmacology of AAVhu68.hGAA and immune responses. **(A)** GAA enzyme activity in plasma, **(B)** anti-hGAA total antibody titers, and **(C)** anti-AAVhu68 NAb titers. Each data point represents an individual animal and timepoint. Nine animals had a negative AAVhu68 NAb baseline while two had pre-existing NAb titers of 1:10 to 1:80 at screening and 1:20 to 1:160 at day 0 pre-dosing. Peripheral blood mononuclear cell (PBMC) ELISPOT responses to **(D)** hGAA peptide pools and **(E)** AAVhu68 peptide pools from eleven rhesus macaques that received IV AAVhu68.CAG.vIGF2.hGAA vectors at 1x10^13^ GC/kg or 5x10^13^ GC/kg or combined IV and ICM at 5x10^13^ GC/kg and 3x10^13^ GC, or at 1x10^14^ GC/kg and 1x10^13^ GC, respectively. Each data point represents a separate peptide pool from an individual animal.

GAA enzyme activity in plasma rapidly increased and reached a maximum level on day 3 (**Figure 1A**). Average peak levels were the highest in NAb-negative animals that received the combined IV/ICM dose (535-fold over baseline for IV 1x10^14^ GC/kg/ICM 1x10^13^ GC and 178-fold over baseline for IV 5x10^13^ GC/kg/ICM 3x10^13^ GC), followed by NAb-negative animals that received the high IV dose 5x10^13^ GC/kg (130-fold over baseline) and NAb-negative animals that received the low IV dose 1x10^13^ GC/kg (23-fold over baseline), respectively. We detected the lowest peak levels in NAb-positive animals, with day-3 levels of 17.9-fold (NAb=1:20) and 4.3-fold (NAb=1:180; **Table S3**) over baseline, respectively. Enzyme activity moderately decreased between days 7 and 14 while remaining above baseline values in all animals. Activity then sharply declined after day 21 as anti-GAA antibody levels increased, with only three animals presenting values above baseline at day 60 (**Figure 1A**, **Table S3**). These three animals, which had GAA activity at 8- to 10-fold over baseline on day 60, also had the lowest level of anti-GAA antibodies (**Figure 1B**, **Table S3**), suggesting that the activity loss in plasma after day 21 was, at least in part, due to anti-drug antibodies. As expected, NAb levels against AAVhu68 rose after AAV administration, and day-60 titers were comparable in all animals, regardless of their baseline titers (**Figure 1C**).

### T-cell immune responses in NHPs following AAV-hGAA gene transfer

IFN-γ ELISPOT assay responses in peripheral blood mononuclear cells were more frequent and of higher intensity in cells stimulated with transgene peptide pools (**Figure 1D**) compared with AAVhu68 capsid peptide pools (**Figure 1E**). Interestingly, hGAA ELISPOT responses showed marked inter-individual variability, ranging from absent in three animals (<55 spot-forming units per million cells) to responses higher than 1,000 spot-forming units per million cells in two animals (both against hGAA peptide pool D). The two NAb-positive animals had low ELISPOT responses to the capsid, similar to NAb-negative animals. Interestingly, the high-NAb-titer animal mounted a robust anti-transgene ELISPOT response despite having very little to no detectable transgene expression in tissues. It is possible that this scenario could involve immune cell transduction following the uptake of opsonized capsid, as shown by the increased biodistribution to the spleen in this animal (**Table S2**). As hGAA peptide pool D seemed to elicit the most robust responses, we performed ELISPOT epitope mapping by stimulating liver lymphocytes isolated from animals 180717 and HS1605511 with peptide sub-pools and then individual peptides. We obtained positive responses to two individual overlapping peptides in each cases, thus narrowing the response to a single hGAA immunodominant peptide sequence in these two animals, which corresponded to hGAA amino acid positions 771–780 in the first animal (TWYDLQTVPI, **Table 1**), and amino acids positions 766-775 in the second (YFPLGTWYDL, **Table 1**). This slight shift in the immunodominant amino acid sequence suggests a hot spot for immunogenicity in the C terminal region of hGAA is located around the “TWYDL” peptide. This hGAA region differs from the rhesus GAA sequence (National Center for Biotechnology Information sequence XP_014975985.2) by three amino acids, L769S, D774N and Q776E.

**Table 1:** ELISPOT response epitope mapping in liver lymphocytes.

Animal	GAA peptide pool	ELISPOT result (liver)	ELIPSOT + GAA sub-pool	Sub-pool peptide positions	ELIPSOT + individual peptide	Immunodominant peptide sequence
180717	D	2,143	D.3	153; **160**; 167; 174; 181; 188; 195	160	YFPLG**TWYDLQTVPI**
			D.4	154; **161**; 168; 175; 182; 189	161	**TWYDLQTVPI**EALGS
			D.9	158; 159; **160**; **161**; 162; 163; 164	160 161	YFPLG**TWYDLQTVPI** **TWYDLQTVPI**EALGS
HS1605511	D	1,673	D.2	152; **159**; 166; 167; 174; 181; 188; 195	159	AEVTG**YFPLGTWYDL**
			D.3	153; **160**; 167; 174; 181; 188; 195	160	**YFPLGTWYDL**QTVPI
			D.9	158; **159**; **160**; 161; 162; 163; 164	159 160	AEVTG**YFPLGTWYDL** **YFPLGTWYDL**QTVPI

Bolded letters indicate immunodominant peptides.

### Safety of AAV-hGAA gene transfer in rhesus macaques

**Figure 2:**
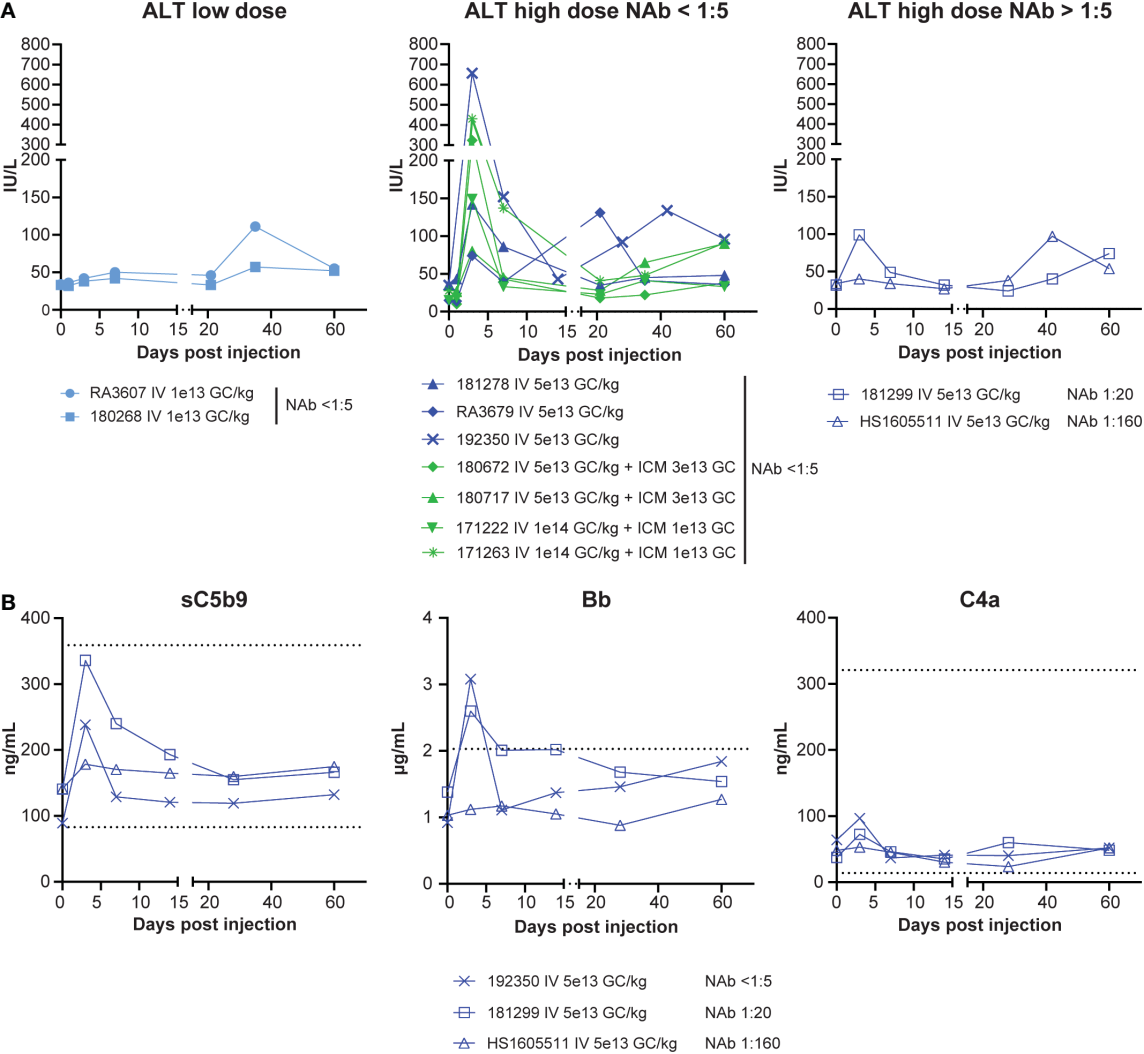
Safety, bloodwork. **(A)** Alanine transaminase (ALT) levels from eleven rhesus macaques that received IV AAVhu68.CAG.vIGF2.hGAA vectors at 1x10^13^ GC/kg (IV low dose) or 5x10^13^ GC/kg (IV high dose) or combined IV and ICM at 5x10^13^ GC/kg and 3x10^13^ GC or at 1x10^14^ GC/kg and 1x10^13^ GC. Data are separated by dose level and the presence/absence of pre-existing NAbs to AAVhu68. **(B)** Complement factors sC5b-9 (terminal complex), Bb (alternative-pathway-specific), and C4a (classical-pathway-specific) from three animals presenting no, low, or moderate NAb titers at baseline. Each data point represents a separate peptide pool from an individual animal.

We did not observe any abnormalities in animals that received the AAVhu68.vIGF2.hGAA test article in terms of cage-side or clinical veterinary observations. Clinical pathology readouts consisted of CBC counts, blood chemistry and electrolyte panels, coagulation panels, and troponin I levels. We measured complement factors sC5b-9, Bb, and C4a in plasma from the NAb cohort to monitor for potential immune-complex-mediated complement activation. We observed test-article-related perturbations for transaminase levels (aspartate aminotransferase and alanine aminotransferase), complement factors, and troponin I. Alanine aminotransferase levels were transiently elevated in all animals that received the high dose IV (>5x10^13^ GC/kg) at day 3, with the exception of the NAb 1:160 animal, which did not demonstrate any acute elevation (**Figure 2A**). We observed a second, mild, non-dose-dependent alanine aminotransferase elevation between days 21 and 42 in fewer animals, likely due to anti-transgene immune responses (**Figure 2A**). Among the three animals that had plasma sampled for complement investigations, the terminal complement factor sC5b-9 was transiently elevated on day 3 in the NAb-negative and NAb 1:20 animals, whereas no activation was observed in the NAb 1:160 animal (**Figure 2B**). This result, together with the fact that the alternative complement (Bb) – and not the classical pathway (C4a) – was activated, suggests that no significant immune-complex-mediated complement activation occurred in our cohort despite the inclusion of animals with pre-existing NAbs to AAV.

### Cardiac toxicity after AAV-hGAA gene transfer in rhesus macaques

**Figure 3:**
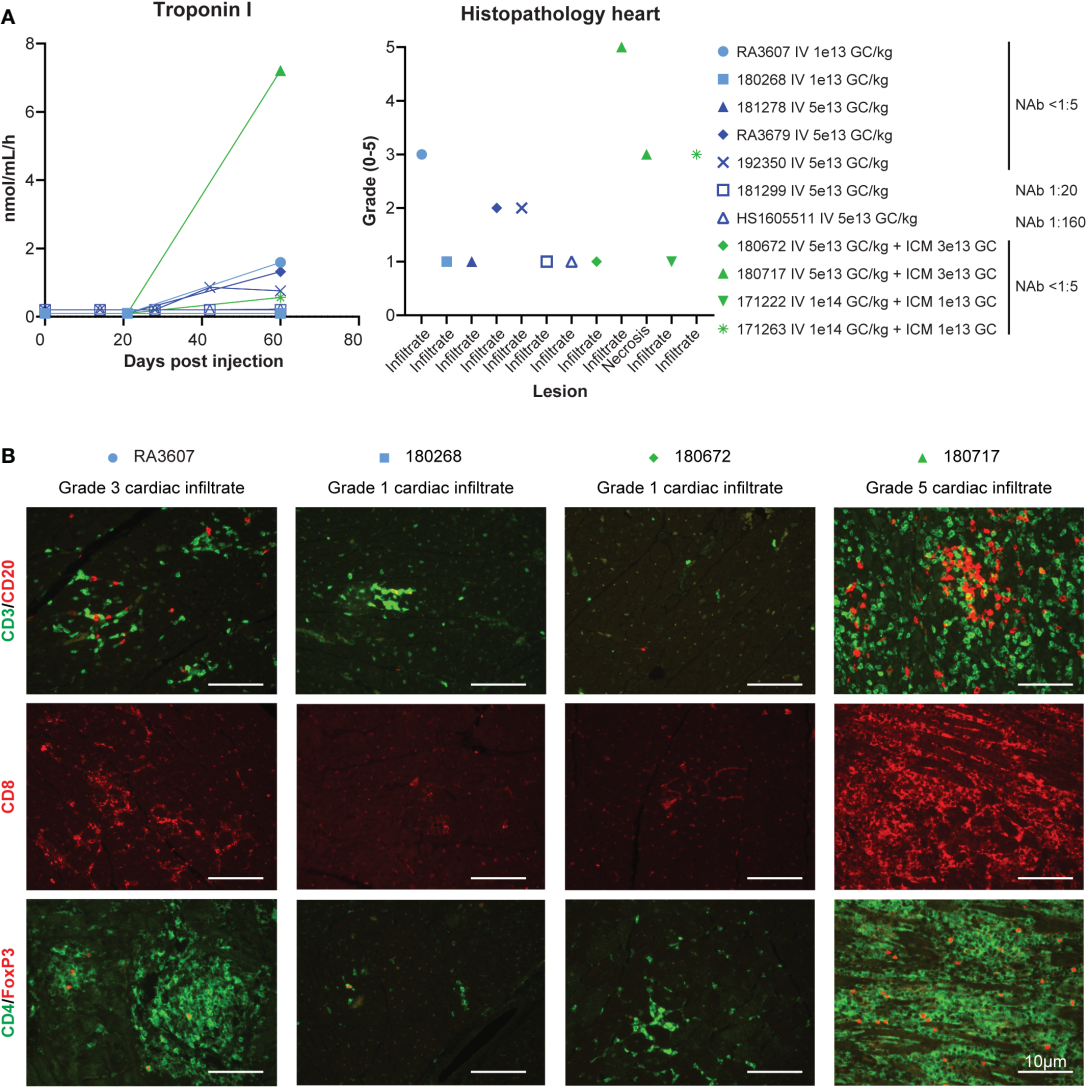
Safety, cardiac toxicity. **(A)** Cardiac troponin I levels and cardiac histopathology from the eleven rhesus macaques presented in the previous figures. Cardiac findings (infiltrate and/or necrosis) were graded by a board-certified veterinary pathologist (ELB) on the following severity scale: 0 (within normal limits), 1 (minimal mononuclear infiltrates, similar to what is commonly observed as background in macaques), 2 (mild), 3 (moderate), 4 (marked), and 5 (severe). When different scores were given to different cardiac regions (ventricle, atrium, septum), the worst score is presented here. For full scoring result see **Table S4**. **(B)** Immunophenotyping of the mononuclear cell infiltrate by immunofluorescence on heart tissue sections *via* T-cell-specific markers.

Troponin I, a biomarker of myocardial damage, was elevated from day 42 to the final timepoint (day 60) in one low-dose and four high-dose animals (**Figure 3A**). This elevation correlated with histopathological analysis of the hearts after necropsy, which showed cardiac infiltrates that exceeded normal background levels in only the five animals that had troponin I elevations (**Figure 3B**). Troponin I levels at day 60 correlated with the severity of histopathology findings; we observed the highest troponin I level of 7.21 ng/mL in the animal with severe (grade 5) infiltrate (180717). This animal was also the only one to present active cardiomyocyte necrosis, and muscle findings with moderate diaphragm inflammation and mild myofiber necrosis. Liver findings were within the expected background range, with minimal-to-mild infiltrates in all the animals. Apart from the heart and muscle, DRG also presented with test-article-related findings of neuronal degeneration ranging from absent in the animals that received the DRG-detargeted construct [same expression cassette with four tandem repeats of miR183 target sites in the 3’ untranslated region, as described in (20)] to marked (grade 4) neuronal degeneration in the two animals that also exhibited the worst cardiac findings (RA3607, 180717; **Figure S3**). There were no brain findings.

**Figure 4:**
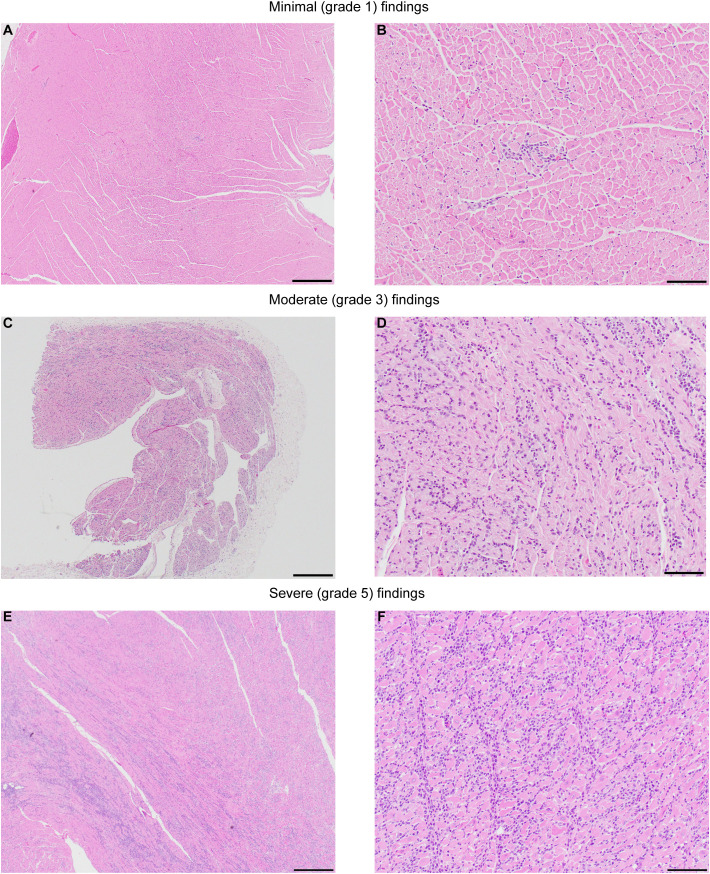
Representative images showing the severity range of microscopic findings in heart. NHPs administered AAVhu68.vIGF2.hGAA at various routes of administration (ROA), including intravenous (IV 5x10^13^ GC/kg) and combination IV (5x10^13^ GC/kg) and ICM (3x10^13^ GC). **(A, B)** animal RA3679, minimal (grade 1) myocardial mononuclear cell infiltrates, left ventricle. **(C, D)** Animal RA3607, moderate (grade 3) myocardial mononuclear cell infiltrates, right atrium. **(E, F)** Animal 180717, severe (grade 5) myocardial mononuclear cell infiltrates with associated fibrosis and moderate (grade 3) cardiomyocyte degeneration and necrosis, septum. The extent of cardiomyocyte involvement in this animal warranted a separate diagnosis. See also **Table S4**. Hematoxylin and Eosin; Scale bar: 4x **(A, C, E)**=500µm, 20x **(B, D, F)**=100µm.

Representative microscopic findings in the heart of NHPs administered AAVhu68.vIGF2.hGAA at various doses showing minimal grade 1 (frequently seen as a background finding in NHPs), moderate grade 3, and severe grade 5 findings are shown in **Figure 4**. The distribution of the findings was consistent across and throughout sections of heart (right and left atria and ventricles, plus septa); however, the findings in a single animal, RA3607, were most severe in the right atrium compared to ventricle (**Table S4**). The findings in animal 180717 involved widespread marked (grade 4) to severe (grade 5) myocardial mononuclear cell infiltrates with associated fibrosis and mild (grade 2) to moderate (grade 3) cardiomyocyte degeneration and necrosis. The extent of cardiomyocyte involvement in this animal warranted a separate diagnosis. Immunophenotyping on cardiac sections showed a florid CD3-positive, T-cell-rich infiltrate with abundant cytotoxic CD8 T cells and few CD4/FoxP3-positive regulatory T cells in the heart that had moderate-to-severe infiltrates, compared with minimal-to-no staining in animals that had normal background infiltrate levels (**Figure 3B**).

The incidence of troponin I elevation and cardiac infiltrates was close to 50% and not dose dependent (one out of two low-dose animals and four out of nine high-dose animals). Cardiac toxicity corresponded to higher ELISPOT responses to hGAA peptide pools in three animals (RA3607, RA3679, 180717; **Figure 1D**). Outliers in this dataset were the NAb 1:160 animal (HS1605511), which presented high-anti hGAA ELISPOT responses but no cardiac toxicity, and very high dose IV 1x10^14^ GC/kg plus ICM 1x10^13^ GC animal 171263 that had moderate cardiac findings with negative ELISPOT responses. This result likely represents the impact of transgene expression levels on the transgene-specific cardiac toxicity. In animal HS1605511, NAbs inhibited cardiac transduction (**Figure S2**), thus preventing cardiomyocytes from expressing the antigenic epitopes. In animal 171263 (IV 1x10^14^ GC/kg and ICM 1x10^13^ GC), very high transgene levels were seen in the heart (**Figure S1**) and in the plasma (highest peak expression, **Table S3**), possibly leading to overexpression-related toxicity, although it is unclear why the other animal from the same dose group had no cardiac findings.

Cardiac troponin I elevations, high ELISPOT responses to hGAA, and CD8-rich cardiac infiltrates are consistent with cardiac toxicity primarily caused by a cytotoxic T-cell-mediated response to the transgene product in transduced cardiomyocytes in three animals from our cohort. We hypothesize that the high inter-individual variability is related to different MHC class I haplotypes in NHPs that may present the hGAA epitopes with more or less affinity. To explore this hypothesis, we sent genomic DNA samples from all of our animals to the Wisconsin National Primate Research Center laboratory for MHC genotyping *via* deep sequencing. The results, presented in **Table S5**, show that the three animals with cardiac toxicity and higher hGAA ELISPOT responses all harbored at least one allele of the Mamu-A002.01 haplotype. The one animal that was homozygous for this allele (animal 180717) presented the most severe troponin I elevation, grade 5 cardiac infiltrate, grade 3 diaphragm infiltrate, grade 4 DRG neuronal degeneration, and the highest ELISPOT responses to hGAA peptides.

## Discussion

T-cell responses to AAV capsids are frequent and have been extensively documented in clinical trials (1, 2, 27). However, T-cell responses to the transgene product following AAV gene therapy have received much less attention and have been primarily documented in Duchenne muscular dystrophy trials (3, 4). Such transgene-dependent responses have the potential to clear transduced cells and reduce therapeutic efficacy, as displayed by two patients who received intramuscular AAV1-encoding alpha-1-antitrypsin. In that case, two patients mounted a cytolytic T-cell response to a common transgene polymorphism not included in the transgene and restricted by a rare MHC I haplotype (28). This potential outcome could negatively impact efficacy (via elimination of transduced cells) and represents a safety concern that was recently highlighted when severe adverse events due to immune-mediated cardiac and skeletal muscle inflammation occurred in several Duchenne muscular dystrophy patients with large N-terminal dystrophin deletions participating in AAV-microdystrophin clinical trials (5).

Responses to non-self epitopes pose a significant challenge when extrapolating safety data from NHPs that receive AAV encoding human proteins. The most common outcome is a mild and non-adverse immune response that is primarily humoral in nature when the AAV encodes a non-self-secreted protein. Other than a consequence on pharmacology readouts (i.e., loss of measurable transgene products in serum due to anti-drug antibodies), these responses are usually non-adverse in NHPs but have been hard to prevent in the long term, even when using strong organ-transplant-like immune suppression regimens (9, 10). It is unclear whether macaque immune responses to non-self epitopes represent an appropriate model for determining potential outcomes in clinical trials enrolling patients with large deletions or null mutations.

In the studies reported here, the nature and severity of anti-transgene immune responses were unusual based on the authors’ experience with NHPs treated with a wide variety of AAV gene therapy products expressing secreted or non-secreted proteins. We have shown that despite high homology between hGAA and rhesus GAA (95% sequence, or 907/952 amino acid identity), several NHPs mounted an adverse cytotoxic response to hGAA, which led to moderate-to-severe myocarditis. The CAG promoter might have contributed to this finding due to its high CpG content and ubiquitous expression. The reason(s) why the heart exhibited the most toxicity and T cell infiltration when transgene expression was also observed in other tissues remains unclear. It might be due to levels of expression, although several animals that exhibited robust cardiac transduction did not develop cardiac toxicity. A possible explanation may come from the MHC class I haplotypes. We further characterized this uncommon immune-mediated cardiac toxicity to demonstrate that an immunodominant epitope in the C-terminal region of hGAA (amino acids 771–780) led to an MHC-I-restricted cytolytic response in the animal that was homozygous for the Mamu-A002.01 haplotype. It is possible that A002.01 could be more robustly expressed on cardiac cells leading to more antigen presentation to CD8 T cells. This allele was present in three out of five animals that presented cardiac toxicity, and in all three animals that had cardiac toxicity with high transgene-dependent ELISPOT responses in our cohort of Chinese-origin rhesus macaques. This allele is not frequent in Chinese- or Indian-origin rhesus or cynomolgus macaques when considering the homologue allele Mafa-A002 (24). Because the rhesus macaque GAA sequence has three polymorphisms compared with the human GAA region that we identified as immunodominant, it is unknown whether this finding would translate to clinical trials that enroll cross-reactive immune-material-positive patients who are presumably tolerant to hGAA. An independent group reported similar observations of troponin I elevations and cardiac inflammation when testing systemic AAV8 expressing hGAA under a skeletal muscle- and cardiac-restricted promoter in cynomolgus macaques (13). Similar adverse findings seen in two different NHP species using a different capsid and promoter highlight the strong immunogenicity of hGAA. The unusual immunogenicity of recombinant hGAA is well known for Pompe patients who receive enzyme replacement therapy, which can lead to robust anti-drug antibody responses that are reportedly associated with the patient’s human leukocyte antigen type (29). Although further studies are required to confirm this association, we propose that the combination of this uncommon macaque MHC I haplotype with the immunogenicity of the hGAA protein and robust cardiac transduction led to test-article-related cardiac toxicity in some animals in our preclinical studies.

Another unexpected and interesting observation made in our NHP cohort was that high-dose systemic AAV (5x10^13^ GC/kg) administration was safe in two animals with pre-existing AAV NAb titers of 1:20 and 1:160, respectively. Transduction levels in the animal with the low titer of 1:20 were high and comparable to those achieved in seronegative animals that received the same AAV dose, whereas gene transfer was mostly inhibited in the animal with the titer of 1:160. We did not observe any adverse events or activation of the classical complement pathway. In previous work published by our laboratory, even the lowest titer of 1:5 led to significant inhibition of liver gene transfer at AAV doses of 6 x 10^12^ GC/kg (30). The present study suggests that the threshold for inhibition might depend on the total AAV dose and that acceptable efficacy and safety can be achieved within a range of low antibody titers. Although this observation is preliminary and relies on only two NAb-positive animals, it warrants further investigation in well-powered studies, as the potential implications for expanding inclusion criteria in systemic gene therapy trials are considerable.

## Data availability statement

The Rhesus macaques (Macaca mulatta) Illumina MiSeq MHC amplicon raw sequence reads can be found in online repositories. The names of the repository/repositories and accession number(s) can be found in the article/**Supplementary Material**.

## Ethics statement

The animal study was reviewed and approved by Institutional Animal Care and Use Committee of the University of Pennsylvania or Children’s Hospital of Philadelphia.

## Author contributions

JH - Conceptualization, supervision, writing-original draft preparation, investigation, visualization; AR – Writing-original draft preparation, formal analysis, investigation; ST - Supervision, resources, writing-review and editing; NM – Supervision, resources, writing-review and editing; CS – Supervision, investigation; AL – Investigation; KL – Investigation; JC - Resources, supervision; ELB – Investigation; CD - Resources, supervision; HY – Investigation; PB - Resources, supervision; JWe - supervision, writing-review and editing; HD – supervision, writing-review and editing; JWi - Conceptualization, supervision, writing-review and editing. All authors contributed to the article and approved the submitted version.
