# Supplementary material for: Immune transgene-dependent myocarditis in macaques after systemic administration of adeno-associated virus expressing human acid alpha-glucosidase
Source: Front Immunol. 2023 Mar 22;14:1094279. doi: 10.3389/fimmu.2023.1094279 (PMC10073725; doi:10.3389/fimmu.2023.1094279)
Supplement: Supplementary file 1 [file DataSheet_1.pdf]

Immune transgene-dependent myocarditis in macaques after systemic administration of adeno-associated-virus expressing human acid-alpha-glucosidase

Supplementary Material

Supplementary Figures

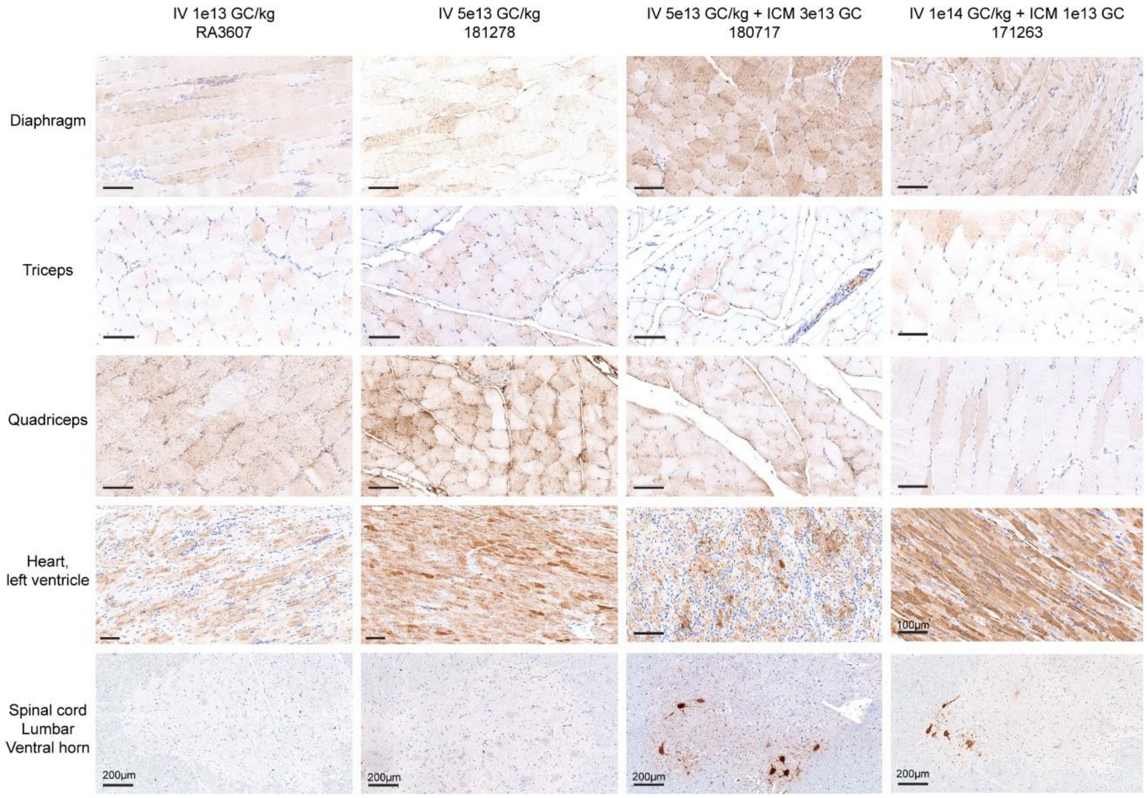

Figure S1. Transgene expression in various tissues. Representative pictures of anti-hGAA IHC stained sections from the diaphragm, triceps brachialis muscle, quadriceps muscle, heart (left ventricle), and lumbar spinal cord ventral horns.

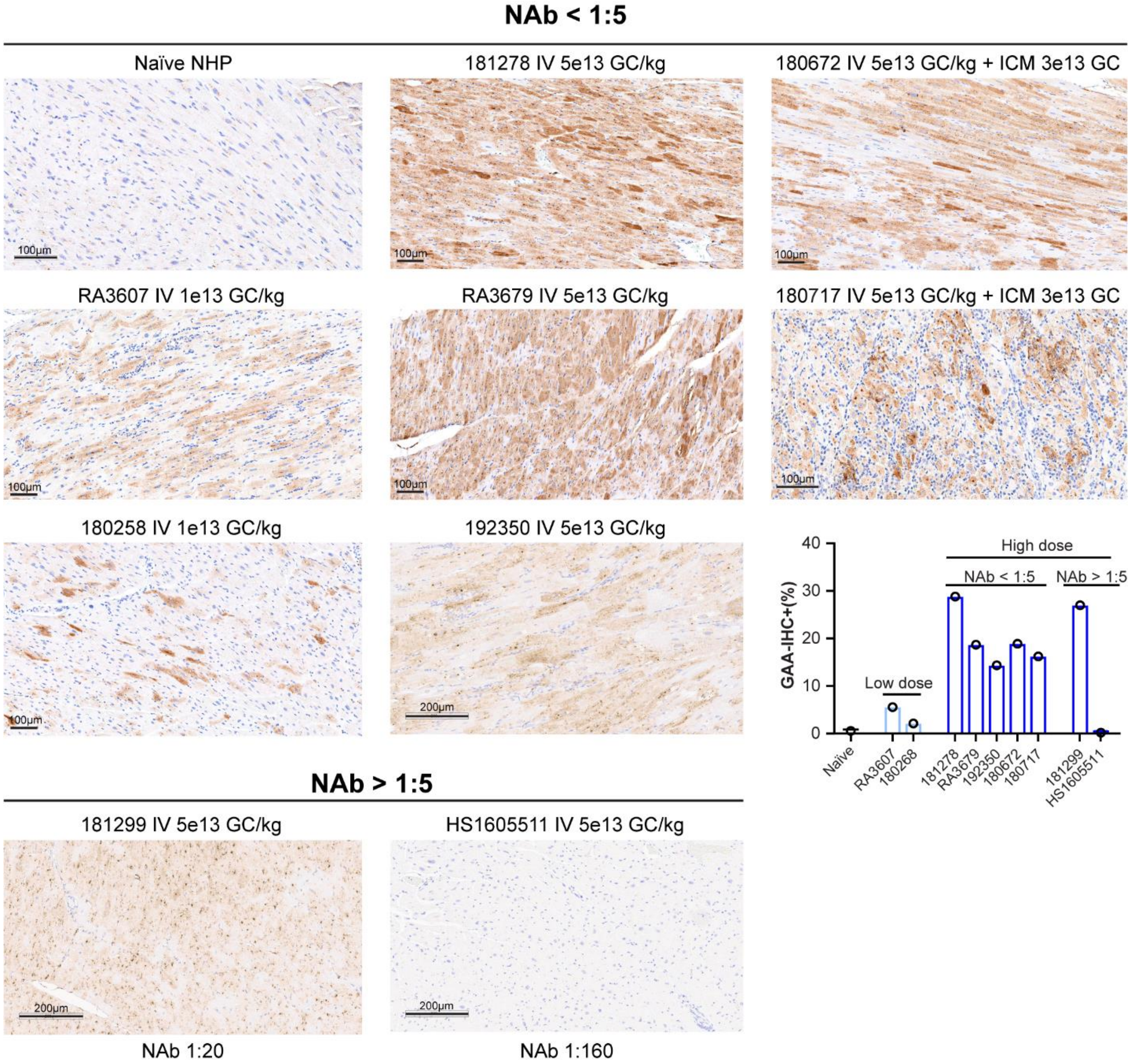

Figure S2. Cardiac transduction. hGAA protein expression in heart tissue, as determined by immunohistochemistry using an anti-hGAA primary antibody that does not cross-react with endogenous macaque GAA, as seen in the naïve NHP control. The graph shows whole-slide quantification of the immunohistochemistry-positive surface area from a left ventricle heart section of each animal.

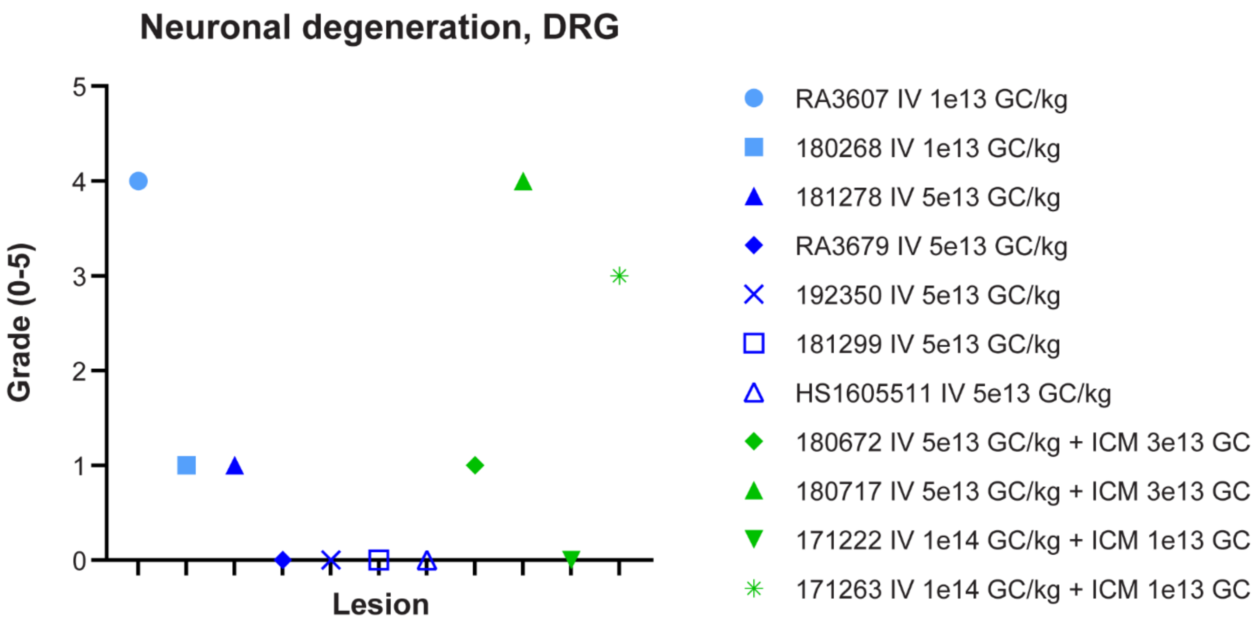

Figure S3. DRG histopathology. DRG findings (neuronal degeneration) were graded by a board-certified veterinary pathologist (ELB) on the following severity scale: 0 (within normal limits), 1 (minimal), 2 (mild), 3 (moderate), 4 (marked), and 5 (severe).

Supplementary Tables

Table S1. Study design

Route	Dose	Baseline NAb	Duration	Vector	Animal ID
IV	1×10^{13} GC/kg	<1:5	60 days	AAVhu68.CAG.vIGF2.hGAAco.rBG	RA3607
		<1:5			180268
IV	5×10^{13} GC/kg	<1:5			181278
		<1:5			RA3679
		<1:5		AAVhu68.CAG.vIGF2.hGAAco.4x-miR183.rBG[C]	192350
		1:10–1:20[A]			181299
		1:80–1:160[B]			HS1605511
IV + ICM	5×10^{13} GC/kg + 3×10^{13} GC	<1:5		AAVhu68.CAG.vIGF2.hGAAco.rBG	180672
		<1:5			180717
	1×10^{14} GC/kg + 1×10^{13} GC	<1:5			171222
		<1:5			171263

[A] Animal 181299: Baseline NAb screening titer 1:10; day of injection titer 1:20

[B] Animal HS1605511: Baseline NAb screening titer 1:80; day of injection titer 1:160

[C] Identical vector except for the 3’ untranslated region containing a four-tandem repeat of a DRG-specific microRNA target site (previously described in (17))

Table S2. Vector genome biodistribution (GC/DG)

Treatment	AAVhu68.CAG.vIGF2.hGAAco.rBG						AAVhu68.CAG.vIGF2.hGAAco.4x-miR183.rBG		
Route, dose	IV, 1 x 10^{13} GC/kg		IV, 5 x 10^{13} GC/kg		IV, 5 x 10^{13} GC/kg + ICM, 3 x 10^{13} GC		IV, 5 x 10^{13} GC/kg		
Animal	RA3607	180268	181278	RA3679	180672	180717	192350	181299	HS1605511
Body weight (kg)	5.44	4.64	4.90	4.26	6.48	6.90	6.94	5.28	8.30
Baseline NAb	< 1:5	< 1:5	< 1:5	< 1:5	< 1:5	< 1:5	< 1:5	1:10–1:20[A]	1:80–1:160[B]
Liver	12.31	12.04	143.69	197.29	97.33	57.93	144.62	76.734	9.14
Spleen	0.07	0.12	1.14	1.14	0.35	0.25	0.09	0.73	5.79
Heart	0.25	0.14	2.81	0.76	0.91	0.23	2.16	3.46	0.08
Quadriceps	0.01	0.01	0.11	0.31	0.14	0.10	0.11	0.18	0.02
Soleus	0.05	0.03	0.41	0.20	0.13	0.28	0.37	0.59	0.05
Tibialis anterior	0.01	0.01	0.08	0.10	0.12	0.06	0.28	0.19	0.02
Biceps brachii	0.02	0.02	0.07	0.24	0.08	0.09	0.63	0.28	0.05

[A] Animal 181299: Baseline NAb screening titer 1:10; day of injection titer 1:20

[B] Animal HS1605511: Baseline NAb screening titer 1:80; day of injection titer 1:160

Table S3. GAA activity and anti-GAA antibody titers in plasma

Animal	GAA activity baseline	GAA activity on day 3	GAA activity on day 7	GAA activity on day 60	Day-60 GAA/baseline	Anti-GAA titer on day 60
RA3607	24.4	280.8	148.1	6.0	0.2	1:2,952,450
180268	8.9	489.7	347.9	5.9	0.7	1:1,968,300
181278	9.5	2,392	1,148.8	78.4	8.3	1:36,450
RA3679	17.3	1,223	270.5	12.6	0.7	1:328,050
192350	6.2	679.9	74.3	3.3	0.5	1:488,000,000
181299	7.5	134.6	69.6	59.8	8	1:781,250
HS1605511	7.0	29.6	20.0	9.5	1.4	1:19,531,250
180672	12.7	3,818.3	1,107.6	7.3	0.6	1:1,968,300
180717	13.4	840	151.7	6.3	0.5	1:2,952,450
171222	3.4	4,188.8	1,179.2	34.5	10	1:109,350
171263	23.6	10,273.6	1,200.8	10.3	0.4	1:984,150

Table S4. Severity and distribution of myocardial findings

Animal ID	Myocardial infiltrates (severity grade)			Myocardial infiltrates/fibrosis (severity grade)			Cardiomyocyte degeneration/necrosis (severity grade)		
	Left	Right	Septum	Left	Right	Septum	Left	Right	Septum
RA3607	2	3*	2	-	-	-	-	-	-
180268	1	1	1	-	-	-	-	-	-
181278	1	1	1	-	-	-	-	-	-
RA3679	1	1	2	-	-	-	-	-	-
192350	2	2	2	-	-	-	-	-	-
181299	1	1	1	-	-	-	-	-	-
HS1605511	1	1	1	-	-	-	-	-	-
180672	1	1	1	-	-	-	-	-	-
180717	-	-	-	5	4	5	3	2	3
171222	1	1	1	-	-	-	-	-	-
171263	3	3	2	-	-	-	-	-	-

Heart sections evaluated included atrium, valve and ventricle from right and left sides and aorta, aortic valve and septum from the section septum. The distribution of the findings was generally consistent across and throughout sections of heart; however, the findings in a single animal (*, see **Fig 4, c-d**) was most prominent in the right atrium compared to ventricle. Severity grading scale: minimal (grade 1), mild (grade 2), moderate (grade 3), marked (grade 4), severe (grade 5).

Table S5. Animals, MHC class I Mamu haplotypes, and correlation with cardiac toxicity

Animal	Species	Gender	MHC A (Class I)		MHC B (Class I)		Cardiac toxicity	hGAA ELISPOT response	Accession number<br><br>(use https://www.ncbi.nlm.nih.gov/biosample)
RA3607	Rhesus macaque	Male	**A002.01**	A008.01	B012.01	B015.01	**Yes**, grade 3	+	SAMN33714804
180268		Male	A008.01	A023.01	B001.01	B028.01	No	-	SAMN33714805
181278		Male	A224.01	A008.01	B069.02	B015.01	No	+	SAMN33714802
RA3679		Female	**A002.01**	A032.01	B012.01	B106.01	**Yes**, grade 2	+	SAMN33714803
192350		Male	A019.01	A056.01	B013.01	B076.01	**Yes**, grade 2	-	SAMN33714810
181299		Female	A032.01	A056.01	B028.01	B003.01	No	-	SAMN33714811
HS1605511		Male	A049.01	A018.02	B047.01	B015.05	No	+	SAMN33714812
180672		Male	A004.01	A023.01	B012.02	B024.01	No	-	SAMN33714806
180717		Male	**A002.01**	**A002.01**	B001.01	B024.01	**Yes**, grade 5	+	SAMN33714807
171222		Female	A018.01	A056.01	B013.01	B076.01	No	-	SAMN33714808
171263		Female	A003.01	A019.01	B039.01	B039.01	**Yes**, grade 3	-	SAMN33714809
